# Formative evaluation prior to implementation of a brief treatment for posttraumatic stress disorder in primary care

**DOI:** 10.1186/s43058-023-00426-2

**Published:** 2023-05-04

**Authors:** Sarah E. Valentine, Cara Fuchs, Elyse A. Olesinski, Natalya Sarkisova, Laura B. Godfrey, A. Rani Elwy

**Affiliations:** 1 Department of Psychiatry, Boston Medical Center, Boston, MA USA; 2 Department of Psychiatry, Boston University Chobanian & Avedisian School of Medicine, Boston, MA USA; 3 Boston University School of Public Health, Boston, MA USA; 4 Department of Psychiatry and Human Behavior, Alpert Medical School of Brown University, Providence, RI USA; 5 Center for Healthcare Organization and Implementation Research, VA Bedford Healthcare System, Bedford, MA USA

**Keywords:** Primary care, Formative evaluation, Posttraumatic stress disorder, Implementation

## Abstract

**Background:**

Successful implementation of evidence-based treatments (EBT) for posttraumatic stress disorder (PTSD) in primary care may address treatment access and quality gaps by providing care in novel and less stigmatized settings. Yet, PTSD treatments are largely unavailable in safety net primary care. We aimed to collect clinician stakeholder data on organizational, attitudinal, and contextual factors relevant to EBT implementation.

**Methods:**

Our developmental formative evaluation was guided by the Consolidated Framework for Implementation Research (CFIR), including (a) surveys assessing implementation climate and attitudes towards EBTs and behavioral health integration and (b) semi-structured interviews to identify barriers and facilitators to implementation and need for augmentation. Participants were hospital employees (*N* = 22), including primary care physicians (*n* = 6), integrated behavioral health clinicians (*n* = 8), community wellness advocates (*n* = 3), and clinic leadership (*n* = 5). We report frequency and descriptives of survey data and findings from directed content analysis of interviews. We used a concurrent mixed-methods approach, integrating survey and interview data collected simultaneously using a joint display approach. A primary care community advisory board (CAB) helped to refine interview guides and interpret findings.

**Results:**

Stakeholders described implementation determinants of the EBT related to the CFIR domains of intervention characteristics (relative advantage, adaptability), outer setting (patient needs and resources), inner setting (networks and communication, relative priority, leadership engagement, available resources), and individuals involved (knowledge and beliefs, cultural considerations). Stakeholders described strong attitudinal support (relative advantage), yet therapist time and capacity restraints are major PTSD treatment implementation barriers (available resources). Changes in hospital management were perceived as potentially allowing for greater access to behavioral health services, including EBTs. Patient engagement barriers such as stigma, mistrust, and care preferences were also noted (patient needs and resources). Recommendations included tailoring the intervention to meet existing workflows (adaptability), system alignment efforts focused on improving detection, referral, and care coordination processes (networks and communication), protecting clinician time for training and consultation (leadership engagement), and embedding a researcher in the practice (available resources).

**Conclusions:**

Our evaluation identified key CFIR determinants of implementation of PTSD treatments in safety net integrated primary care settings. Our project also demonstrates that successful implementation necessitates strong stakeholder engagement.

Contributions to the literature

- Determined the need for brief, tailored, PTSD therapies in safety net primary care.
- Used developmental formative evaluation guided by the Consolidated Framework for Implementation Research to understand factors that may influence successful implementation of a brief PTSD treatment in safety net integrated primary care.
- In preparation for PTSD treatment implementation in integrated primary care, stakeholders suggested the refinement of detection and referral procedures; interdisciplinary trainings; dual capacity building in non-specialty and specialty care clinics; revising hiring practices; and offering provider incentives for evidence-based treatment delivery.

## Background

Safety net settings provide health care services to patients regardless of their ability to pay [1], and play a major role in serving populations with low access to care, including low-income and racial and ethnic minoritized groups. Posttraumatic stress disorder (PTSD) disproportionately affects patients seen in safety net hospitals, with prevalence estimates as high as 46% [2–5]. Despite high prevalence, PTSD is under-recognized and undertreated, with only 13% of individuals with PTSD receiving care [3]. Evidence-based treatments (EBTs) for mental health, including PTSD, are largely unavailable in safety net settings and, when available, are often exclusively provided in specialty care. However, low provider adoption of EBTs in specialty care settings is associated with structural challenges, such as high workload demands, little protected time for training, and attitudinal barriers [6, 7]. Additionally, patients’ perceptions of lower-quality care [8] and experiences of mental health stigma, discrimination [9–12], and medical mistrust [11] have resulted in underutilization of specialty care. For these reasons, patients in low-resource settings may prefer care in less stigmatized non-specialty settings (e.g., primary care) [13–17]. Gaps in access to EBTs have driven large racial and economic disparities [8, 18–20].

Integrated behavioral health (IBH) models utilize collaboration between behavioral health professionals and physicians to provide comprehensive medical and behavioral health care [21]. Successful implementation of EBTs in integrated primary care may address treatment access and quality gaps by expanding access and providing care in a less stigmatized setting [12, 14]. However, there may be unique challenges to implementation in primary care due to competing clinical and administrative priorities in interdisciplinary settings, and brief treatment and referral models of care [22]. EBTs for PTSD have only recently been adapted for primary care, though these data are limited to Veterans Health Administration (VHA) settings [23–26], including some with specialized, residential PTSD programs [27]. Given the complexities of implementation, it is essential to engage stakeholders in system and treatment redesign and in developing plans to promote adoption and sustainability of EBTs for PTSD. Possemato and colleagues (2018) detailed how stakeholder-engaged formative evaluation was essential in successful EBT implementation within VHA primary care [28], yet given differences between the VHA and safety net hospitals, there is a need to further optimize and test these EBTs in integrated primary care clinics within safety net hospitals [29, 30].

Implementation is a complex process that requires information from all organizational levels to develop and modify an intervention for system integration [31, 32]. Developmental formative evaluation can be used to optimize EBTs prior to their enactment to maximize the likelihood of success by identifying anticipated barriers and facilitators to implementation and tailoring interventions to meet the needs of individuals and organizations involved [31–34]. The parent study will test the effectiveness and implementation outcomes of a brief PTSD treatment in safety net integrated primary care, adapted for the local setting and population based on the stakeholder data reported here. We used a concurrent mixed methods developmental formative evaluation [33], which relies on a data-driven, learning-oriented approach to assess program and organization priorities related to EBT implementation. Our developmental formative evaluation assessed (a) drivers of current and ideal practices for PTSD treatment in primary care (e.g., patient screening, clinical objectives for quality and care); (b) facilitators and barriers to intervention adoption in this setting (guided by the Consolidated Framework for Implementation Research [CFIR] [35]); and recommendations on how to address these barriers/facilitators. Our finalized implementation blueprint that specifies expert-recommended implementation strategies [36] derived from this developmental formative evaluation is published elsewhere [37].

## Methods

### Setting

This study took place in the largest safety net hospital in New England, with hospital-based integrated primary care clinics serving approximately 50,000 patients. Most patients (70%) are insured by Medicaid (public health insurance for those requiring financial assistance), and over half (56%) of Medicaid-insured patients at the hospital are in need of behavioral health services. As such, the hospital has adopted an IBH model, which relies on collaboration, coordination, and colocation of primary care physicians (PCPs) and behavioral health specialists. PCPs are represented by residents and physicians. The IBH team includes clinical social workers and psychologists (7.0 FTE) and psychiatrists (1.5 FTE). IBH therapists have 50% of their time reserved for 30-min scheduled visits. The remaining time is unscheduled to allow for warm handoffs. The small number of IBH clinical staff relative to needs of the population underscores the need for low-intensity, time-limited interventions, and highly accessible stepped-up care when referring out. As such, IBH uses a stepped care approach to ensure that patients are offered the most effective, yet least intensive, care first, and treatment is “stepped-up” to higher level care when needed. The hospital also employs community wellness advocates, trusted members of the community, to support high-risk patients via advocacy, education, and care coordination.

### Selected intervention

Our parent study will inform the development of a stepped care approach to PTSD treatment that spans primary care and speciality care. The central aim is to optimize and pilot test a “step one” intervention in safety net integrated primary care. Skills Training in Affective and Interpersonal Regulation (STAIR) [38–41], recently adapted for use in VHA primary care (STAIR-PC) [23], was selected in consultation with IBH leadership as an appropriate step one intervention in primary care during the study planning period due to its brevity and low intensity. STAIR-PC is a five-session cognitive behavioral therapy for PTSD that includes psychoeducation and coping skills training to address emotion regulation and interpersonal difficulties common in trauma survivors [23]. STAIR is an empirically supported treatment [38, 39], including in safety net settings [40] and when delivered by non-specialists [41], and STAIR-PC has demonstrated preliminary effectiveness [23], including when delivered by paraprofessionals (peers) [42].

At the time of this study, there were no published data to inform expected response rates for this protocol, yet studies published using a similar protocol have since found clinically meaningful change in 67% of participants (peer-delivery) [42], and in VHA samples, 13–46% retained their PTSD diagnosis [23, 43]. The parent trial will assess not only PTSD symptom reduction, but also whether engagement in a “step one” intervention may improve subsequent engagement in mental health services (e.g., by reducing mental health stigma and other barriers).

### Participants

Participants were hospital employees in integrated primary care, selected to represent the distinct roles in patient care and potential different viewpoints on implementation determinants. Primary care participants included primary care providers and leadership, and IBH participants included *potential* interventionists in the future trial (IBH therapists, community wellness advocates) and behavioral health leadership. Participants were recruited at clinical team meetings and by email. We first recruited our potential interventionists, provided an orientation to the intervention, and then used purposive sampling to recruit additional key informants nominated by primary care and behavioral health leadership, for a total of 22 participants. Participation was voluntary and included 1 h of time, between online surveys and an in-person semi-structured interview with [S.E.V.]. Surveys varied by role, with some specific to potential interventionists (see Table 1). Data were collected from October 2018 to February 2019. Participants were remunerated $20. This study received an exempt determination from the Institutional Review Board.

**Table 1:** Descriptive information on participant roles and study measures completed

Participants	*N*	Study role	Measures completed
**Primary care**			
Primary care physicians (PCPs)	6	Stakeholder	ICS, LIM, Semi-structured interview
Primary care leadership	3	Stakeholder	
** Total**	**9**		
**Integrated behavioral health (IBH)**			
Behavioral health leadership	2	Stakeholder	ICS, LIM, EBPAS-15 Semi-structured interview
IBH therapists	8	Potential interventionist	
Community wellness advocates	3	Potential interventionist	
** Total**	**13**		

*ICS* Implementation climate scale [44], *LIM* Levels of integration measure [45], *EBPAS-15* Evidence-Based Practice Attitude Scale [46], *IBH* Integrated behavioral health

We used a concurrent mixed-methods formative evaluation, where qualitative and quantitative data are collected in parallel, and analysis for integration of these data begins after data collection for both methods has been completed [47]. Following guidelines, we analyzed our two forms of data separately and then merged these data to present a convergent, integrated overview. Our evaluation was guided by the CFIR [35] to characterize the implementation determinants of STAIR-PC in a safety-net IBH setting. CFIR is one of the most widely used frameworks in implementation science and allowed us to identify indicators of *anticipated* implementation outcomes (i.e., predictors of future implementation success/failure), which would inform adaptations prior to trial start that would maximize actual implementation success [48]. We assessed four core CFIR domains to examine implementation determinants of a brief cognitive behavioral therapy for PTSD: (1) *intervention characteristics*, (2) *inner setting,* (3) *outer setting*, and (4) *characteristics of individuals involved* (Table 2). Two survey measures were used to assess the CFIR inner setting domain: (1) the implementation climate scale (ICS) [44] consists of 6 items (from 0 – “not at all” to 4 – “to a very great extent”), which assess level of agreement with statements reflecting three organizational climate dimensions of innovation use: expected, supported, and rewarded. We used the ICS to characterize the level of support for implementing EBTs for PTSD in primary care; and (2) the Levels of Integration Measure (LIM) [45] measures the integration of care between IBH clinicians and PCPs across 6 domains, including integrated clinical practice, systems integration, training, relationships, shared decision making, beliefs and commitment, and leadership (from 1 – “strongly disagree” to 5 – “strongly agree). We utilized the LIM to understand commitment to the IBH model, which we anticipated may be a predictor of later implementation. The ICS and LIM were completed by all participants.

**Table 2:** Interview guide based on selected CFIR constructs

**Intervention characteristics**
**Adaptability**
*What do you think it will take to deliver a 5-session structured cognitive-behavioral intervention in the integrated primary care setting?*
*What are your concerns about this? Do you think it will work, or not work? What do you think you need to make a 5-session cognitive behavioral therapy intervention work?*
**Outer setting**
**Patient needs and resources**
*How well do you think a cognitive behavioral therapy skills intervention would meet the needs of the individuals served by your organization?*
*How well do you think a brief PTSD intervention would meet the needs of the individuals served by your organization?*
*What barriers do you think the individuals served by your organization might face in order to participate in the intervention?*
**Inner setting**
**Compatibility**
*How do you think a 5-session cognitive behavioral therapy intervention fits in with your organization?*
*How does the intervention potentially NOT fit?*
*When thinking about a 5-session cognitive behavioral therapy intervention, how well do you think this might fit in with your organization’s current work flow processes?*
* Does it fit with the way you currently deliver care (for example, your collaborative care model)?*
*What are some issues or complications that you think may arise?*
^a^**Characteristics of individuals**
**Knowledge and beliefs about the intervention**
*What do you know about this brief PTSD intervention?*
*Of these things that you know, what makes you think that this intervention will work, or not work, in your setting?*
*How do you feel about the intervention being used in your setting?*
*How do you feel about the goal of implementing the intervention in your setting?*
*When you first think about this brief PTSD intervention, what are the first thoughts or feelings that you have about it?*
**Self-efficacy**
*How confident are you that you will be able to use the intervention?*
*What gives you that level of confidence (or lack of confidence)?*

*CFIR* Consolidated Framework for Implementation Research [35], *PTSD* Posttraumatic stress disorder

^a^These questions were only asked to integrated behavioral health therapists

We used the Evidence-Based Practice Attitudes Scale (EBPAS-15) [46] to assess the CFIR domain of characteristics of the individual. This 15-item survey assesses the level of agreement with four dimensions of attitudes towards EBTs generally (not specific to STAIR-PC): (1) intuitive appeal of EBTs, (2) likelihood of adopting EBTs, given requirements, (3) openness to new practices, and (4) perceived divergence of usual practice. Scores range from 0 – “not at all” to 4 – “to a very great extent” to indicate how strongly an attitude is held. The EBPAS-15 was completed only by IBH participants (our potential interventionists and two stakeholders from behavioral health leadership).

A qualitative interview guide was developed in consultation with our primary care community advisory board (CAB). The CAB (*N* = 9) includes hospital employees in integrated primary care with decision-making authority and highly relevant expertise. The CAB is involved in all phases of the parent study, including interpretation of findings from this formative evaluation and subsequent implementation blueprint development. Full detail on our approach to CAB engagement is published elsewhere [37]; to reduce burden on participants, we worked with the CAB to select only interview questions from the CFIR Interview Guide Tool (https://cfirguide.org/guide/app/#/) deemed most relevant to the local setting. See Table 2 for the full interview guide. Interviews were completed by all participants. Only primary interventionists were asked to comment on appropriateness of the candidate intervention. Although none had delivered STAIR-PC, most therapists were familiar with cognitive behavioral therapy.

### Data analysis

We ran frequencies and descriptives of survey data to characterize the overall implementation climate and attitudes in the local setting and conducted post hoc analyses to assess differences between participant type (IBH vs. primary care) and survey responses.

For qualitative data, we applied directed content analysis [49], which started with an a priori coding framework derived from CFIR but allowed for the addition of emergent codes during the codebook development and refinement process. We used a two-phase process for codebook development, consisting of a team-based approach [50] to analyzing interview data. Three team members coded data in phase 1 and 4 members in phase 2. Phase 1 of codebook development utilized a rapid coding procedure [51] based on audio review of interview data. The purpose of the rapid coding procedure was to quickly identify major ordinate themes that could be presented to CABs to operationalize adaptations to STAIR-PC prior to trial start [37].

Phase 2 of codebook development was a conventional approach [49], wherein coders were able to expand, refine, and finalize the codebook prior to coding of interviews that had been transcribed verbatim by study staff. The coding team met weekly to mitigate bias, discuss differing perspectives, and improve inter-coder reliability. The final coding phase consisted of repeating this process until no new codes emerged from the data, and consensus was reached. After finalizing the codebook, transcripts were double-coded using NVivo 12 software (QSR International) until a benchmark of at least 20% of the interviews with inter-coder agreement above 80% was met (i.e., 6 double-coded transcripts attained 97% inter-coder agreement). Then, the remaining transcripts were equally divided among the two primary coders for independent coding. The team met weekly during independent coding to ensure ongoing consensus.

### Data integration

We use the Journal Article Reporting Standards (JARS) for Mixed Methods Research [52] to guide reporting. In our concurrent mixed methods approach [47], data integration occurred in two ways: (1) through a connecting method, as our survey data links with the qualitative data through our purposive sampling of primary care and IBH stakeholders [47], and (2) through a joint display approach to synthesize a correlation between our quantitative data (“low” or “high”) and responder sentiments described in the interview (“positive” or “negative”).

Our joint display is presented in Table 4. The qualitative data provided a prospective analysis of specific CFIR constructs pertaining to (a) drivers of current and ideal practices; (b) facilitators and barriers to adoption of an intervention. Quantitative data were embedded within qualitative findings for each CFIR domain and construct to describe the assessment of specific organizational and attitudinal factors within respondants’ narrative descriptions.

## Results

Our developmental formative evaluation involved 22 participants, including 11 potential interventionists (8 IBH therapists, 3 community wellness advocates) and 11 PCPS and clinic leaders and administrators (6 PCPs, 5 clinic leaders and administrators representing psychology, psychiatry, social work, and operations). No participants refused to participate or dropped out. Sociodemographic information was not collected to preserve employee privacy.

### Surveys

Table 3 provides descriptives of survey findings. Overall, participants endorsed moderate organizational support (CFIR inner setting) for implementation of evidence-based practices (ICS M = 2.09, SD = 0.81). Participants reported low ratings on the ICS subscales staff selection (M = 1.78, SD = 1.05) and rewards (M = 0.85, SD = 0.71), suggesting that although participants favorably rated their organization’s attitudinal support of evidence-based practices, the organization does not explicitly seek to hire staff who are trained in or value evidence-based practices (CFIR characteristics of individuals involved), nor does the organization provide financial incentives for adoption of these approaches (CFIR inner setting). Among IBH participants (*n* = 13), there was moderately strong attitudinal support for adoption of EBTs (EBPAS-15 M = 2.80, SD = 0.72), with ratings comparable to those observed among community mental health service providers [46, 53]. Overall, participants reported slightly positive attitudes towards behavioral health integration in the clinic (LIM M = 3.51, SD = 0.37). Importantly, mean and standard deviation for survey scores did not significantly vary between primary care and IBH clinician participants for the ICS (PCP 37.67 (9.19); IBH 36.85 (6.67); p = 0.82) and LIM (PCP 121.44 (9.34); IBH 122.85 (14.86); p = 0.79), emphasizing that priorities for program implementation for PTSD were similar across stakeholder type. Indeed, a joint display comparison of high and low survey scores shows shared sentiments across both primary care and IBH individuals from the ICS and LIM (Table 4).

**Table 3:** Descriptive data from surveys

Measure	Response format	Item content	Subscale	M (SD)
*All Participants (N* = *22)*				
Implementation climate scale (ICS) (5 items, 0–4 scale)	Evaluative (not at all to a very great extent)	Assessment of agreement with statements reflecting three organizational climate dimensions of innovation use (answered by those in primary care and IBH)	Focus on EBT	2.78 (0.62)
			Educational support for EBT	2.59 (0.94)
			Recognition for EBT	2.22 (0.85)
			Rewards for EBT	0.85 (0.71)
			Selection for EBT	1.78 (1.05)
			Selection for openness	2.41 (0.81)
			**Overall**	**2.09 (0.81)**
Levels of integration measure (LIM) (5 items, 1–5 scale)	Evaluative (strongly disagree to strongly agree)	Assessment of integration between PCPs and IBH clinicians (answered by those in primary care and IBH)	Integrated clinical practice	3.47 (0.62)
			Systems integration	3.23 (0.38)
			Training	3.20 (0.71)
			Relationships	3.76 (0.60)
			Shared decision making	3.26 (0.63)
			Beliefs and commitment	4.23 (0.45)
			Leadership	3.70 (0.89)
			**Overall**	**3.51 (0.37)**
*IBH-level participants (N* = *13; 11 potential interventionists, 2 behavioral health leadership)*				
Evidence-Based Practice Attitude Scale (EBPAS-15) (15 items, 0–4 scale)	Evaluative (Not At All to A Very Great Extent)	Assessment of agreement with attitudes towards EBTs (answered by those in IBH)	Requirements	2.64 (1.34)
			Appeal	3.04 (1.08)
			Openness	2.67 (0.72)
			Divergence	2.81 (0.74)
			**Overall**	**2.80 (0.72)**

*PCP* Primary care physician, *IBH* Integrated behavioral health, *EBT* Evidence-based treatment. Joint display of survey results with qualitatively derived codes for evaluation of integration and organizational climate among primary care and IBH

**Table 4:** Quotes from participants corresponding with high and low survey scores

Survey rating	Clinician type	Examples
**Treatment plans for PTSD to meet patient needs** Knowledge and training for identifying trauma and PTSD; treatments systematically meet the needs of patients		
High ICS Scores	IBH	“When we [IBH] go through the psychiatric review of symptoms, I always ask in the intake, ‘is there a traumatic event that you witnessed that is still upsetting you today?’”
Low ICS scores	Primary care	“I think one of the challenges is that I don’t find that my colleagues in primary care are particularly trauma-literate, so I really believe that we have to do some more education for other staff in our clinic.”
	Primary care	“I have to say, my familiarity with PTSD is not as strong.”
**Support for evidence-based practices** Training, workshops, or materials about applying EBT for treatment; positive attitudes and champions for evidence based practice within clinics; integration between primary care and IBH		
High LIM scores	Primary care	“I think the community health workers would be very enthusiastic about it [manualized PTSD therapy].”
Low LIM scores	IBH	“I think that we [IBH] are very siloed out in family medicine, kind of out of people’s radar.”
	Primary care	“Much of the energy has been focused on meeting what we [primary care] have to do…to deliver [PTSD] program[s] in the eyes of [state insurance programs]. There’s a tremendous amount of work left to be done, like optimizing care and delivery.”

*PTSD* Posttraumatic stress disorder, *IBH* Integrated behavioral health, *ICS* Implementation climate scale [44], *LIM* Levels of integration measure [45], *EBT* Evidence-based treatment. Joint display that relates categorical survey results from ICS and LIM to quotes from primary care and IBH stakeholders

### Interview findings

Although our interview guide focused on determinants of STAIR-PC, responses were often pertinent to the overall provision of brief mental health interventions in the integrated primary care setting, and not specific to PTSD treatment. See Table 5 for more detail on CFIR constructs (based on the original CFIR) that we identified as influencing implementation, and initial recommendations generated through interviewee feedback and CAB engagement.

**Table 5:** Factors that influence implementation of an EBT for PTSD in safety net primary care

CFIR constructs	Definition	Examples
**Intervention characteristics**		
Relative advantage	Stakeholders’ perception of advantages of implementing the five-session, manualized intervention versus an alternative solution	Positive attitudes about the intervention compared to alternative EBTs (brevity, flexibility, transdiagnostic application, effectiveness) and compared to current standard of care (primarily psychoeducation, relaxation training only) Although some patients may need higher intensity therapies, this is an effective lower intensity treatment which may have engagement advantages
Adaptability	The degree to which an intervention can be adapted, tailored, refined, or reinvented to meet local needs	Interventions needs to fit current practice, including session length and scheduling frequency
**Outer setting**		
Patient needs and resources	The extent to which patient needs, as well as barriers and facilitators to meet those needs, are accurately known and prioritized by the organization	May experience challenges with prioritizing PTSD-focused treatment in the context of social determinants of health and related barriers to engagement (transportation, caregiving responsibilities, socioeconomic status) *Emergent theme:* Mental health stigma, language, literacy, care setting preferences, patient treatment priorities, especially in context of social determinants, were mentioned as important considerations
**Inner setting**		
Networks and communications	The nature and quality of webs of social networks and of formal and informal communications within an organization	Challenges with internal and external referral processes for behavioral health treatment and the inability of PCPs to access therapy notes have led to issues with care coordination Challenges unique to a teaching hospital, where staff rotate and turnover frequently, have resulted in problems with communication. Need for ongoing and repeated training and communication about treatment options and clinic processes
Relative priority	Individuals’ shared perception of the importance of the implementation within the organization	ACO has led to organizing around depression care management; PTSD is not currently a quality metric. This is both a barrier and facilitator, since some of the improvements are good for all mental health services (e.g., routine depression screening)
Leadership engagement	Commitment, involvement, and accountability of leaders and managers participating in the implementation	Need to gain support from operations managers and population health leaders in the practice for implementation success, especially in regard to protecting time and modifying workflows to accommodate EBTs
Available resources	The level of resources dedicated for implementation and on-going operations, including money, training, education, physical space, and time	By nature, IBH prioritizes access (brief screening, treatment referral); however, accommodating routine appointments typical for EBTs can be a challenge Due to time and resource burdens, screening is not feasible in most PCP or IBH therapist visits. Need to consider the role of medical assistants in initial screening for PTSD Difficulties securing protected time for training and consultation Need to embed researcher on clinical support time for training purposes and to respond to implementation challenges in real-time
**Characteristics of individuals**		
Knowledge and beliefs about the intervention	Individuals’ attitudes toward and value placed on the intervention as well as familiarity with facts, truths, and principles related to the intervention	PCP knowledge gap of best practices for assessment, treatment options, and referral process for PTSD (e.g., rely on heuristics of stereotypes to determine who needs assessment; asking too much detail about trauma events in screening process; lack of knowledge of referral pathways) PCPs unsure of their ability to assess for PTSD. Express concerns about distressing patients and not knowing how to respond or support PCPs expressed positive attitudes about co-learning with IBH therapists and how this project may foster improvements in collaborative care Most IBH therapists expressed that trauma-focused cognitive behavioral therapy is a good fit for their patients, especially if it can be delivered in a brief format Some IBH therapists expressed attitudes that brief treatments are not appropriate for PTSD (requires long-term therapy)

*CFIR* Consolidated Framework for Implementation Research [35], *EBT* Evidence-based treatment, *PTSD* Posttraumatic stress disorder, *ACO* Accountable care organization, *IBH* Integrated behavioral health, *PCP* Primary care physician

### CFIR: intervention characteristics

#### Relative advantage

Respondents affirmed that a brief five-session cognitive behavioral therapy intervention was appropriate for use in primary care and was considered a more advantageous intervention than current practices for several reasons. An IBH provider shared that, relative to first-line EBTs for PTSD which are longer and more intensive, the structure of a brief manualized treatment would help orient patients to the short-term approach to PTSD therapy while also providing effective care: *“if we’re [IBH] already planning for five sessions, then we [IBH] can market it [manualized PTSD intervention] that way for patients when they come in for the intake… plus, manualized treatments are super convenient.”*

They expressed how low-intensity and brief treatment in IBH may be more suitable for patients seen in primary care, and especially for patients with barriers to engagement in high-intensity specialty care (e.g., stigma, mental health literacy, affective avoidance, or suppression). They hypothesized that patients may be more open to engage in stepped-up care (e.g., first-line EBTs for PTSD) if needed after STAIR-PC, as one PCP stated:* “[for] people who will never ‘land’ in [specialty care], if we give this [manualized PTSD treatment] and they have a good experience, they may be more willing to do the next level.”*

#### Adaptability

Respondents shared how the proposed intervention could fit the local practice with some modifications. As one PCP noted, *“[physicians] want this [type of therapy]. [When we] have a very short [and] effective way to treat PTSD — where we teach people these skills they can practice at home to reduce their symptoms — [PCPs] can [offer this treatment option] and keep an eye on [treatment progress].”* One IBH provider described how STAIR-PC was an appropriate fit based on patient needs and the IBH setting: *“I think [a manualized PTSD treatment] is in line with the other interventions that we are trying to offer in IBH. So I think [the treatment fits the setting] completely. Both within an IBH model and for our patient population, where I think, you know, our focus tends to be on how do we help improve their functioning right now in their lives? So I think it fits within the scope of the work that we’re doing, and I don't see any reason why it wouldn’t be a good fit for this patient population.”*

### CFIR: outer setting

#### Patient needs and resources

Respondents noted that socioeconomic determinants of health were common barriers to engagement in and prioritization of PTSD treatment. As one IBH provider explained, *“[engagement] could be challenging given the complexity of working with [patients experiencing] …homelessness or financial instability.”* Additionally, respondents described transportation and caregiving barriers that contribute to patients’ late or missed appointments, resulting in less provider contact and high patient dropout. As one PCP emphasized,* “the biggest thing for a lot of patients is competing priorities, living in a world where finding a job and being safe is the first priority and convincing people that it [mental health] is worth their time [comes second].”*

##### Emergent themes

Several themes regarding cultural considerations emerged as a subconstruct within this CFIR domain, reflective of the need for increased considerations of race and racism, stigma, shame related to mental health care, and language and literacy barriers among patients with PTSD. Respondents described how patients may be hesitant to disclose trauma and seek treatment for PTSD due to mental health stigma and stigma of referral to specialty care. As one community wellness advocate shared, *“the word [name of specialty care clinic] is very stigmatized.”* Respondents felt that a stepped care approach to PTSD treatment, with triaged levels, may help patients engage at their preferred setting and intensity of treatment. Further, respondents hypothesized that stigma may be a reason to expect better engagement in primary care settings (v. specialty mental health) for the proposed 5-session intervention.

Providers additionally described the role of racism in the patient-provider relationship. As one community wellness advocate stated: *“a lot of patients have mentioned to me, especially if [they’re] a person of color, [that] they [patients] want to be able to relate [to] and see someone of color.”* This perspective highlights how systems in which providers do not reflect diversity of patients may face additional challenges earning trust and engaging patients.

### CFIR: inner setting

#### Networks and communications

Respondents emphasized the importance of care coordination, including the need for clear internal and external referral processes between clinicians within the primary care setting.

##### Referrals to IBH

The process for referring to IBH utilized warm handoffs. Several mentioned that, while ideal, warm handoffs can be logistically difficult: *“I think there’s a kind of subtle disincentive to use [a warm handoff] in that no matter how fast it [a warm handoff] is, you’re grabbing a room and you’re taking time, [an] extra 10 or 15 min to have that warm handoff take place.”* Despite these challenges, the majority of providers supported the use of warm handoffs and recognized their role in improving patient engagement. As another PCP explained, *“the opportunity to do long warm handoff[s] will definitely increase that [patient] buy-in.”* Respondents reported that the practice of relying on patients to spontaneously disclose trauma history or PTSD symptoms during routine primary care visits may miss patients with mild-to-moderate PTSD symptom severity or those who are reluctant to disclose. The high severity referrals received by IBH, and access issues when attempting to refer out to specialty care led some respondents to believe that patients seen in IBH may be higher in symptom severity than typically seen in IBH models of care.

##### Referrals to specialty care

By contrast, the referral process to specialty care is less specified and rarely utilizes warm handoffs. IBH respondents voiced reservations about referring out to specialty care, noting concerns about patient dropout or uncertainty about current therapies offered in specialty care. One IBH provider described the siloed nature of IBH and specialty care: *“I’ve only ever been to [the specialty care clinic] once or twice, and I don’t know that many people who work there, so…I have very little idea what actually goes on over there.”*

#### Relative priority

At the time of this evaluation, the hospital was shifting to an accountable care organization (ACO), which links reimbursements to quality metrics and reductions in the cost of care. Some respondents felt that the central focus on ACO quality metrics may pose challenges to adding a screener for PTSD. Under the ACO, depression care screening is prioritized, and hospitals are evaluated, scored, and provided funding based on the frequency and consistency of Patient Health Questionnaire (PHQ-2 and -9) administration. Other respondents felt positively that the ACO shift may be a stepping stone to improving PTSD outcomes. One PCP noted how the redesigned system prompted by the ACO may allow greater access to behavioral health services, stating, *“one of the real opportunities [of] the ACO is in restructuring the way that we think about [the] clinic more broadly and more aggressively than we thus far have.”*

#### Leadership engagement

Importantly, respondents raised the need to have regular communication with between clinical leaders and team members about different resources. As one PCP noted, *“there’s a lot going on, and…. [as part of the leadership team] … I have a good handle of all the programs …but, I think I'm probably the minority. The PCPs who are there once a week may not know of all the resources that are available to our patients.” *Additionally, respondents noted that buy-in more broadly from operations managers and population health leaders was critical to successful implementation, including securing protected time and resources to support adoption.

#### Available resources

Workflow and productivity demand, documentation and case management tasks, and administrative meetings create extensive time pressures on all providers that limit availability for trainings needed for successful implementation. Respondents emphasized the need for additional protected time for training and suggested, in the meantime, that researchers capitalize on existing protected times for training.

Respondents also identified the availability of appointments for active, ongoing therapy in IBH as a major barrier to PTSD intervention implementation. Furthermore, rescheduling missed appointments in a timely fashion is challenging due to high patient volume and low efficiency. Respondents emphasized the need to address these workflow challenges to accommodate EBT scheduling demands (at least two visits per month).

Respondents also noted the importance of embedding the researcher (study principal investigator) in the practice, with one IBH therapist stating, *“I would find [it] really helpful [to have the principal investigator embedded in the clinical support team] because …if there’s anything wrong or different later [that impacts the roll-out], …that could help keep the momentum going.”*

### CFIR: characteristics of individuals involved

#### Knowledge and beliefs about the intervention

Respondents described the need for educational trainings tailored to PCPs and IBH clinicians to improve understanding of trauma, PTSD diagnosis, and effective treatments. As one IBH provider expressed, *“providers… just don’t know what to do with trauma.”*

##### PTSD assessment

Respondents explained how some providers applied variable heuristics and clinical judgment to decide which patients to screen for trauma and PTSD. PCPs described gathering a thorough trauma history with full details of index events, which is not the standard (or advised) process for screening. Others described screening for PTSD symptoms only when PCPs knew or suspected trauma exposure based on voluntary disclosure or patient characteristics (race and immigration status). As one PCP shared: *“the only people I know [who] have PTSD are the ones who volunteer it [trauma history or diagnosis].”* Respondents also described how some PCPs assign a PTSD diagnosis based on trauma exposure alone, without directly assessing for symptoms. Still, other PCPs may avoid asking patients about trauma due to concerns around distressing patients or insufficient time to respond to anticipated distress.

##### Current treatment options

PCPs also described lack of knowledge on the types of treatments available via each referral pathway, partly due to lack of access to therapy notes in the electronic medical record. PCPs shared their general enthusiasm for co-learning with their IBH colleagues and how this project might help them to stay abreast of PTSD treatment options.

### Data integration

See Table 4 for our joint display presentation of findings and exemplar quotes. In terms of treatment plans for PTSD to meet patient needs, knowledge of and prioritization of trauma in case conceptualization and treatment planning was reflected in high scores on the ICS, whereas lack of knowledge in trauma assessment and treatment, which was particularly a challenge among primary care providers compared to IBH clinicians, was evident in low ICS scores. Enthusiasm for evidence-based practice across primary care and IBH is consistent with high LIM scores, however, challenges (low LIM scores) were attributed to lack of clinic integration and a focus on abiding by state insurance programs.

## Discussion

Our developmental formative evaluation to support the implementation of an EBT for PTSD in safety net primary care illuminated our understanding of current practices and attitudes and identified facilitators and barriers to implementation. Overall, respondents were optimistic that offering a brief, low-intensity treatment for PTSD in primary care may fit the practice and patients’ desires to receive care in a less stigmatized environment, though they also recognized non-specific challenges to patient engagement, including mental health stigma, medical mistrust, and competing clinical priorities (hierarchy of needs; e.g., housing). Although there was strong attitudinal support for EBTs, cross-cutting time and resource constraints posed major barriers to implementation. Respondents pointed to various system-alignment challenges that would need to be addressed prior to roll out — workflow and scheduling challenges and the need to address training gaps across provider types to support diagnosis, treatment, and referral processes for PTSD. Notably, our survey results also highlighted that the local setting has not prioritized hiring staff trained in evidence-based practices and that current providers have low self-efficacy for treating PTSD. This is one place where safety net settings may contrast with the VHA, which has made access to training in EBTs for PTSD readily available [54]. Improving access to training is one important avenue to address when preparing safety net systems for delivering PTSD treatment.

Our findings extend the literature in identifying PTSD treatment implementation barriers specific to safety net integrated primary care settings. We found organizational support for EBTs was similar or higher than ratings of implementation climate observed in other mental health [44] and substance use settings [55]. Consistent with research on determinants to implementation of EBTs for PTSD across settings [2, 6, 22, 56], important factors included organizational/leadership support for EBTs, clinic operations, intervention feasibility in the practice, provider attitudes towards EBTs, therapist time and capacity, and patient engagement (which may be particularly challenging in low-resource settings due to competing social needs [6]). However, extant research has primarily focused on higher-intensity, burdensome interventions (e.g., cognitive processing therapy, prolonged exposure) in specialty care settings. Unique challenges in primary care include lack of PCP knowledge of PTSD treatment options and lack of brief EBTs for PTSD that are conducive with primary care models [22]. In a review of early research on primary care-based PTSD interventions, Possemato (2011) found promising preliminary effectiveness of brief interventions, yet few have been successfully adopted in usual primary care, and implementation process outcomes are scarce [22]. Our CFIR-guided formative evaluation helps to position these brief treatments within a continuum of care and highlights the critical need to align systems prior to implementation, such as setting up clear pathways for patients to move through the safety net system and receive efficacious treatments at multiple points of service.

One observation we made in conducting this formative evaluation is how important inner setting constructs are to provider buy-in and adoption [56]. For example, PCPs and leadership were more receptive to screening when treatments were readily available in IBH (as part of the same project), and IBH therapists were more receptive to delivering EBTs when there were also efforts to address workflow challenges and simultaneously build capacity in specialty care. The latter is beyond the scope of this project, but has become a focus of departmental initiatives aimed at therapy capacity building across the continuum of care.

### Limitations

Given our use of purposive sampling in this study, results are limited in their generalizability or reproducibility. Furthermore, findings may have been biased by our efforts to focus mainly on barriers (rather than facilitators) to implementation, in order to develop strategies to overcome these. Respondents were also instructed that they could talk about their own practice or the modal practice in their local setting — this again may have biased findings. Our sample included many champions or people with particular knowledge about PTSD. Their views may not reflect the attitudes of all providers in the practice, demonstrating the need for practice-wide assessments of implementation factors. That said, few respondents had prior experience with manualized treatment for PTSD, and therefore responses about CFIR domains were often generalities about behavioral health treatment or cognitive behavioral therapies–-not specific to STAIR-PC. Post-trial data will help us better understand the acceptability of STAIR-PC specifically, as therapists and other stakeholders become more familiar with the intervention as they apply it to their patients in usual care.

We focused the formative evaluation on hospital employees’ perspectives because we anticipated that system alignment challenges would be paramount, yet we acknowledge that patient perspectives are missing. In addition to engaging our primary care CAB, we will also be engaging a patient CAB in the next phase of the parent study, where we develop our implementation blueprint to guide the clinical trial; and we will be conducting exit interviews with trial participants to gain their perspectives on the intervention and implementation. Recognizing the siloed nature of IBH and specialty care, IBH respondents proposed improvements in assessment, screening, referral processes, and treatment available in specialty care. Our data fell short of explicating cross-clinic barriers to implementation that could be identified through the use of process mapping [57].

### Future steps

Respondents noted how patients of color may have greater mistrust of providers due to past negative interactions with health care, including poor quality of care, discrimination, and historical maltreatment of minoritized people. These findings suggest that additional engagement supports, such as effort to improve mental health literacy, reduce stigma, and gain trust are warranted. We propose that engagement of peers or community wellness advocates in this role may be appropriate [58]. Additionally, respondents acknowledged the need to consider variability in language and literacy, as many patients do not speak English as their primary language or are illiterate. Although our formative work suggested that no modifications to core components of the intervention were warranted [37], we identified the need to expand the scope of clinician consultation in the trial to support culturally responsive application of PTSD treatment. For example, our group consultation model will encourage discussions around integrating social contextual factors in case conceptualization and exploring the impact of racism on stress. To ensure that engagement barriers related to racism and stigma are addressed thoroughly, we will conduct exit interviews with providers and patients to assess the need for cultural adaptations to intervention components and will use ongoing CAB engagement to ensure fit with the patient population. We will also seek to expand the intervention with adaptations for patients with advanced age or disabilities. Additional detail on our formative work on racism as an implementation consideration is published elsewhere [59].

## Conclusion

Successful implementation of evidence-based PTSD treatments in safety net hospitals necessitates a strong implementation blueprint [36] or toolkit [60] that includes multi-level interventions and protocols (e.g., detection and referral procedures, integration and collaboration, training programs for various provider types and settings, dual capacity building in non-specialty and specialty care clinics, hiring practices, provider incentives, allocated time for training and consultation) [61]. Indeed, clinic operations that are committed to learning from point of care patient interactions and incorporating a continuous improvement and data-driven process within the delivery and sustainability of EBTs for PTSD have been identified as an important determinant in reach of EBTs within either a primary care or mental health care setting [62]. The heavy lift of successful implementation further illustrates the need for institutions to invest in sustainably embedding implementation scientists within clinical practice [63–65].

## Data Availability

Not applicable.

## Abbreviations

PTSD: Posttraumatic stress disorder
CFIR: Consolidated framework for implementation research
EBT: Evidence-based treatment
PCP: Primary care physician
IBH: Integrated behavioral health
STAIR-PC: Skills training in affective and interpersonal regulation for primary care
ICS: Implementation climate scale
LIM: Levels of integration measure
EBPAS: Evidence-based practice attitudes scale
CAB: Community advisory board
ACO: Accountable care organization
PHQ: Patient health questionnaire
VHA: Veterans health administration
